# Predictors of aspiration, lower respiratory tract infection, and respiratory failure among individuals with Rett Syndrome: analysis of real-world claims data in the United States

**DOI:** 10.3389/fped.2025.1681103

**Published:** 2025-10-20

**Authors:** Krithika Rajagopalan, Nazia Rashid, Dilesh Doshi, Daksha Gopal

**Affiliations:** ^1^ Anlitiks Inc., Windermere, FL, United States; ^2^ Medical Affairs, Acadia Pharmaceuticals Inc., San Diego, CA, United States

**Keywords:** Rett Syndrome, predictors, aspiration, respiratory outcomes, cough, lower respiratory tract infection, respiratory failure

## Abstract

**Background:**

Rett (RTT) syndrome, a rare, neurodevelopmental disorder affects multiple organ-systems (i.e., gastrointestinal, respiratory), with diverse clinical manifestations. While gastrointestinal manifestations are well-known, respiratory manifestations [i.e., aspiration, lower respiratory tract infection (LRTI), and respiratory failure (RF)] and associated predictors are not well-studied. This real-world data analysis evaluated the predictors of aspiration, LRTI, and RF among RTT individuals in the United States.

**Methods:**

A retrospective database analysis using IQVIA's Anonymized Patient Level database from 08/01/2020 to 03/31/2023 was conducted to identify newly diagnosed RTT individuals with ≥1 RTT diagnostic claim (ICD-10-CM: F84.2) between 02/01/2021 and 03/31/2022. Index date was the first RTT diagnostic claim. Eligible sample included individuals with 6-months pre-index and 12-months post-index follow-up, as well as no pre-index cerebrovascular disease or brain trauma diagnosis. Predictors of aspiration, LRTI, and RF were separately evaluated using exploratory backward selection models followed by confirmatory multivariable logistic regressions and reported using odds ratios (OR) with 95% confidence intervals (95% CI).

**Results:**

Of the 1,994 with RTT, 7.27% (*n* = 145), 9.48% (*n* = 189), and 10.08% (*n* = 201) experienced post-index aspiration, LRTI, and RF, respectively. Significant predictors for aspiration were cough [3.39 (1.82–6.29)], dysphagia [3.04 (1.86–4.99)], LRTI [2.34 (1.22–4.51)], and neurological disorders (i.e., epilepsy/convulsions) [1.77 (1.18–2.66)]; LRTI were respiratory disorders [4.06 (2.63–6.27)], RF [3.02 (1.65–5.53)], neurological disorders [1.57 (1.07–2.29)], infections [1.69 (1.04–2.80)], and gastrointestinal disorders [1.57 (1.04–2.37)]; RF were LRTI [4.70 (2.58–8.58)], respiratory disorders [3.38 (2.23–5.13)], dysphagia [2.73 (1.73–4.31)], gastrointestinal disorders [2.09 (1.40–3.10)], musculoskeletal disorders [1.86 (1.01–3.40)], and neurological disorders [1.69 (1.16–2.45)]. Confirmartory models showed similar results.

**Conclusion:**

Baseline neurological and respiratory disorders were common predictors of aspiration, LRTI, or RF. Additional predictors included gastrointestinal disorders for LRTI and RF; and musculoskeletal disorders for RF only. These real-world findings can help inform evidence based clinical decision-making for management of RTT.

## 1 Introduction

Rett Syndrome (RTT) is a rare genetic neurodevelopmental disorder that arises from *de novo* loss-of-function mutations in the methyl-CpG-binding protein 2 (MECP2) gene (1, 2). While it is widely assumed to be diagnosed among females, male children who have the typical MECP2 mutations have also been diagnosed with RTT syndrome (3), a condition typically diagnosed around three years of age in the United States (4). Affecting approximately 1 in 10,000–20,000 live female births worldwide, RTT is one of the leading genetic causes of severe intellectual disability in females (5). Deficiency in MECP2 disrupts normal neuronal development and plasticity, leading to impaired gross and fine motor skills, loss of verbal communication with limited non-verbal abilities, behavioral challenges, hand stereotypies, seizures, and gastrointestinal issues (3, 6). These multisystem comorbidities associated with RTT contribute to substantial morbidity, mortality, and overall healthcare utilization.

While some individuals with RTT live into middle age or beyond, respiratory complications including pneumonia or lower respiratory tract infections (LRTI) among individuals with RTT often lead to recurrent aspiration and respiratory failure (RF), and consequently, reduced life expectancy (7). Given this, respiratory complications have been implicated as the leading causes of health care resource use burden and mortality in RTT, with LRTI (37%), aspiration and/or asphyxiation (32%), and RF (14%) being the three most common causes of death (8, 9). Several studies have reported that pneumonia, aspiration, and respiratory failure are major causes of deaths in RTT cohorts, with mortality rates ranging from 26% to 58% (10, 11). Clinical manifestations include erratic breathing, recurrent apneas, obstructive and central sleep apnea, and chronic aspiration-related complications. Given the severity of respiratory manifestations among individuals with RTT, it is critical to understand the risk factors contributing to these manifestations. In fact, current guidelines emphasize proactive, multidisciplinary management including systematic respiratory surveillance, swallowing assessments, reflux monitoring, and timely interventions such as non-invasive ventilation to reduce morbidity and improve quality of life (12).

The potential interaction between the different comorbidities among individuals with RTT, including breathing disturbances, dysphagia, epilepsy, and scoliosis exacerbate the risk of aspiration and LRTI (11, 13–15). Among these conditions, scoliosis which affects almost three-quarters of individuals with RTT by age 15 can impair posture, mobility, as well as digestive and respiratory functioning (16). On the other hand, epilepsy has been associated with an increased risk of respiratory infections in individuals with intellectual disabilities (15, 17); with aspiration during or after seizures further increasing vulnerability to respiratory infections. Furthermore, poor muscle tone and oromotor non-coordination contribute to feeding difficulties, placing the airway at risk of aspiration, necessitating enteral feeding or gastrostomy (14, 18).

Notwithstanding the manifestations mentioned above, no quantitative studies have been conducted to understand the predictive risk factors associated with respiratory outcomes such as aspiration, LRTI, and RF among RTT. To address this gap, a real-world data analysis was conducted to evaluate risk factors associated with respiratory outcomes (i.e., aspiration, LRTI, and RF) among individuals with incident RTT in the United States, with the goal of informing clinical care and risk mitigation strategies.

## 2 Methods

### 2.1 Study design & data source

A retrospective cohort study was conducted using the IQVIA Anonymized Patient Level Data (APLD) from August 1, 2020, through March 31, 2023 (i.e., study period). The APLD is an open-source, administrative claims data that includes pre-adjudicated, de-identified healthcare claims data for over 130 million beneficiaries in the United States. The database contains detailed information on demographics, medical claims such as diagnosis, procedures, services, and pharmacy claims, making it a robust real-world data resource to complete the study objectives.

### 2.2 Study population

**Figure 1:**
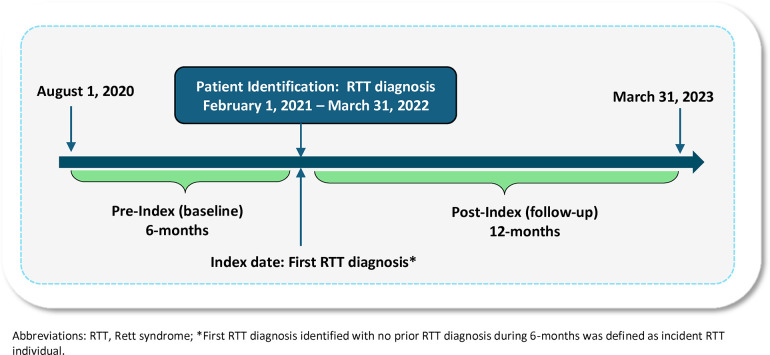
Study design and study population.

The study population identified from the APLD included newly diagnosed RTT individuals with ≥1 inpatient or outpatient diagnostic claim for RTT (International Classification of Diseases, 10th Revision, Clinical Modification (ICD-10-CM: F84.2) in any diagnostic position (primary, secondary, etc.) during the patient identification period from February 1, 2021, through March 31, 2022 (Figure 1). The index date was defined as the first diagnostic claim for RTT (RTT diagnosis date). Eligible patients had no prior history of RTT during the 6-months baseline (i.e., pre-index), thus representing newly diagnosed RTT individuals. Based on the clinical diagnostic criteria of RTT and to exclude potentially those who are misdiagnosed, individuals with diagnostic claims for cerebrovascular disease (ICD-10-CM: I60–I69) or brain trauma (ICD-10-CM: S06) during the baseline period were excluded (19). Lastly, the final study population was required to have a minimum of 6-months of continuous enrollment prior to the index date (baseline period) and a minimum of 12-months of follow-up (post-index).

### 2.3 Study variables

The independent variables in this study included demographic variables such as age, gender, and region. Age was categorized into pediatric (<18 years of age) and adult (≥18 years of age) groups, while gender was recorded as male or female. The regions were classified into four categories: Midwest, Northeast, South, and West. Baseline comorbidities were assessed as the percentage of individuals with at least one ICD-10-CM based diagnostic claim (in any position of claim) during the baseline period; all corresponding ICD-10-CM codes are reported in Supplementary Tables S1, S2. While LRTI was defined by diagnosis codes indicating infections of the bronchi and lungs; RF was identified through codes related to acute and chronic conditions reflecting the severity of respiratory compromise in individuals with RTT. Other important baseline comorbidities included vomiting, cough, and dysphagia. Additionally, we also characterized the percentage of patients who had differential diagnosis of any one or more of the following: autism spectrum disorder, cerebral palsy, non-specific developmental delay, Angelman syndrome, and other childhood disintegrative disorders during the baseline period (See Supplementary Table S2).

Core features of RTT, baseline comorbidities, clinical outcomes of interest, and their associated diagnostic codes are outlined in Supplementary Tables S3, S4. The core RTT features included neurodevelopmental disorders (i.e., behavioral disorders and disturbance symptoms, loss of acquired communication skills, loss of acquired motor skills, prominent hand apraxia/dyspraxia, and others). Baseline comorbidities included: gastrointestinal disorders (i.e., constipation, diarrhea, gastroesophageal reflex disorder, and others), growth abnormalities/nutritional disorders (i.e., underweight, short stature, and others), musculoskeletal disorders (i.e., scoliosis, kyphosis and other spinal deformities), respiratory disorders (i.e., asthma, atelectasis, COPD, and breathing irregularities), neurological disorders (i.e., epilepsy, convulsions), and infectious/viral conditions (i.e., fever, upper respiratory infection, and COVID-19).

#### 2.3.1 Respiratory outcomes & associated predictors

Proportion of patients with ≥1 diagnostic claim for aspiration, LRTI, and RF outcomes in any diagnostic position (primary, secondary, etc.) during the 12-months follow-up were estimated. Aspiration was defined using diagnostic codes (ICD-10-CM: J69.0, J69.8, Y84.4) associated with aspiration pneumonia or other forms of aspiration. LRTI was defined using ICD-10-CM codes for pneumonia, bronchitis, or other forms of pneumonia due to infections (ICD-10-CM: J13.X-J18.X, J20.X, J22.X, J40.X). Similarly, RF was defined using ICD-10-CM codes related to RF (ICD-10-CM: J96.XX, J95.82X, Z99.11, V46.1X, V46.2, Z99.81, R09.2, R09.02). These codes can be found in Supplementary Table S1.

### 2.4 Statistical analysis

Demographics, clinical characteristics, other study measures, and outcome variables were reported descriptively as frequencies and percentages for categorical variables; mean, standard deviation (SD), median, and interquartile range (IQR) were reported for continuous variables. Among individuals with any of these three different respiratory outcomes of interest, mean and median time from incident RTT diagnosis to the first claim for aspiration, LRTI, or RF event during post-index was also calculated. Predictors of the three respiratory outcomes were also separately evaluated through a two-step analytic process: (i) exploratory backward selection logistic regression models and (ii) confirmatory multivariable logistic regression models.

In the first step, three separate stepwise logistic regression models using backward selection were used to identify potential baseline predictors of post-index aspiration, LRTI, or RF, respectively. The dependent variable for each model was the occurrence of post-index aspiration (yes/no), LRTI (yes/no), and RF (yes/no), respectively; the independent variables for each model included age, gender, and 13 potential baseline factors (aspiration, LRTI, RF, vomiting, cough, dysphagia, neurodevelopmental disorders, gastrointestinal disorders, growth abnormalities/nutritional disorders, musculoskeletal disorders, infections/viruses, respiratory disorders, and neurological disorders). Since the goal was to explore external predictors of the outcome of interest, we did not include the baseline occurrence of each of these outcomes, respectively, as they would be heavily correlated with the post-index outcome. However, a sensitivity analysis was conducted in which the baseline occurrence of the outcome of interest, respectively, were also included in the models.

The backward selection logistic regression approach was chosen to allow the model to systematically identify and retain the most impactful predictors and avoid overfitting by sequentially removing the variables with the least explanatory power one after another, based on Akaike Information Criterion (AIC). After the removal of each potential predictor variable with the least explanatory/predictive power, the model was re-evaluated using the AIC to compare the models and ensure the best fit. The final three models retained only those predictors that contributed to the likelihood of aspiration, LRTI, or RF, respectively.

During the second step, three different confirmatory multivariable logistic regression models were conducted to assess the magnitude of association between baseline predictors identified from the exploratory backward selection models and post-index outcomes (aspiration, LRTI, and RF) after adjusting for age and gender. Results of the multivariable logistic regression models were reported as adjusted odds ratios (ORs) and 95% confidence intervals (CIs), with statistical significance determined using *p*-value <0.05. All statistical analysis were conducted using Anlitiks proprietary RapidAnalyzer™ analytic platform that is powered by SQL and RStudio.

## 3 Results

### 3.1 Study population

**Figure 2:**
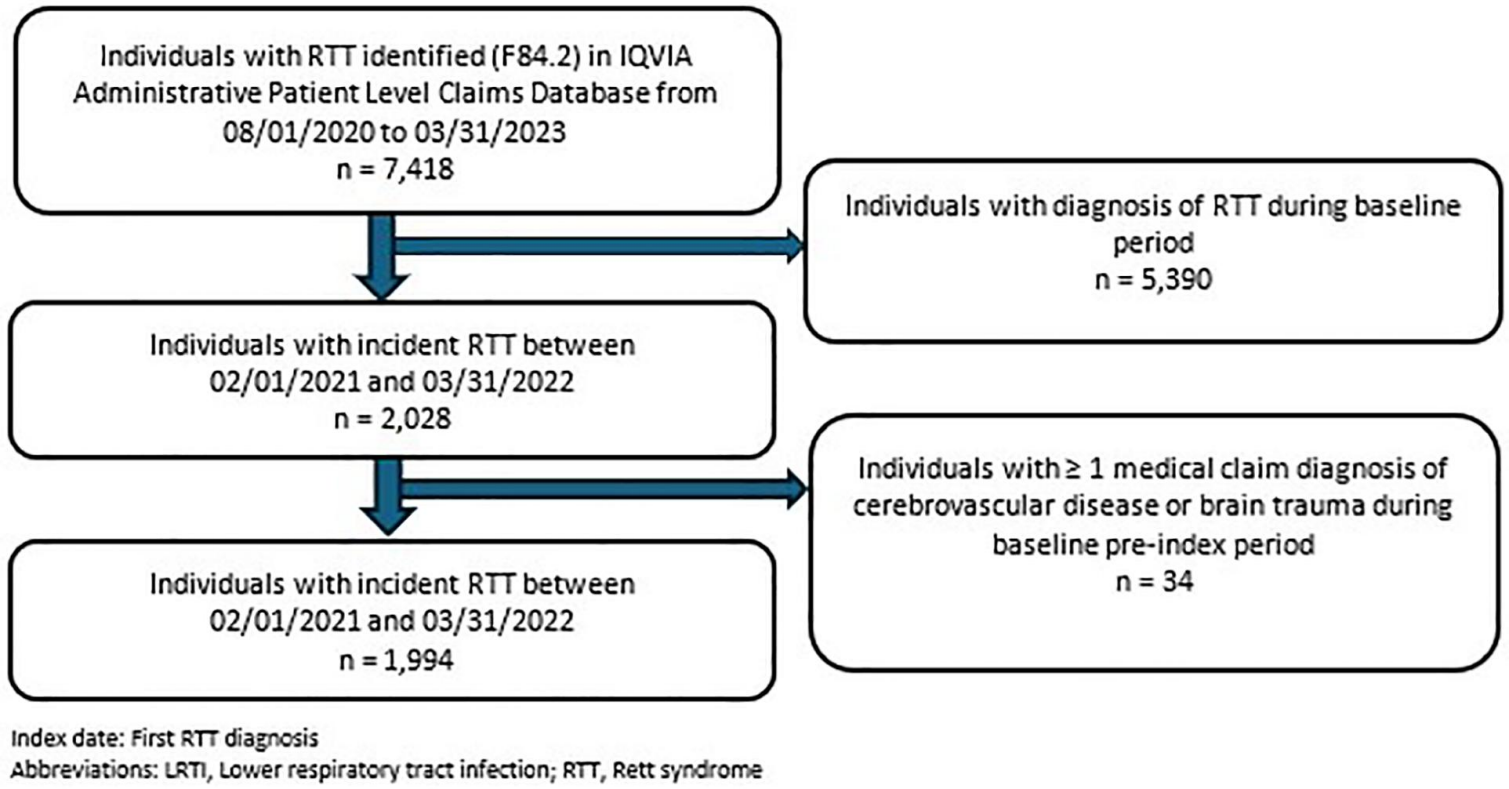
Cohort attrition flow diagram.

A total of 7,418 individuals with RTT were identified in the IQVIA APLD database; of these, 27% (*n* = 1,994) formed the eligible study population as newly diagnosed RTT individuals (Figure 2). Over 75% (*n* = 1,575) of the RTT cohort were female, with 51% (*n* = 1,019) < 18 years of age. The mean age at index date was 21.23 ± 17.7 years. Baseline demographic, clinical, and comorbidity characteristics were categorized into three study outcomes of interest during the post-index period: aspiration, LRTI, and RF status, respectively (Tables 1, 2). Among patients who had aspiration, 16.55% had cerebral palsy and 28.28% had developmental delay, compared to 7.36% and 6.87%, respectively, among those without aspiration (*p* < 0.0001). Similarly, individuals with LRTI vs. without-LRTI had higher rates of cerebral palsy (17.99% vs. 6.98%, *p* < 0.0001) and developmental delay (20.63% vs. 7.15%, *p* < 0.0001). For those with RF vs. without RF, cerebral palsy was present in 17.91% vs. 6.92% without LRTI (*p* < 0.0001) and developmental delay in 18.91% vs. 7.25%, without LRTI (*p* < 0.0001). Autism spectrum disorder was less common among those with LRTI (7.41% vs. 14.46%, *p* = 0.0103) and respiratory failure (4.98% vs. 14.78%, *p* < 0.0001) (Table 1).

**Table 1:** Baseline demographics, differential diagnosis and study outcomes of incident RTT individuals.

Baseline demographics	Aspiration outcome during post-index			LRTI outcome during post-index			Respiratory failure outcome during post-index		
	Yes	No	*p*-value	Yes	No	*p*-value	Yes	No	*p*-value
	*n* = 145	*n* = 1,849		*n* = 189	*n* = 1,805		*n* = 201	*n* = 1,793	
Age, years, mean ± SD	21.66 ± 20.72	21.20 ± 16.35	0.7944	23.13 ± 20.53	21.04 ± 16.24	0.1730	23.85 ± 21.20	20.94 ± 16.10	0.0606
Age, years, *n* (%)									
Pediatric [<18 years]	82 (56.55)	937 (50.68)	0.2017	98 (51.85)	921 (51.02)	0.8887	105 (52.24)	914 (50.98)	0.7908
Adult [≥18 years]	63 (43.45)	912 (49.32)	0.2017	91 (48.15)	884 (48.98)	0.8887	96 (47.76)	879 (49.02)	0.3556
Gender, *n* (%)									
Female	109 (75.17)	1,466 (79.29)	0.2514	148 (78.31)	1,427 (79.06)	0.8828	150 (74.63)	1,425 (79.48)	0.1314
Male	36 (24.83)	378 (20.44)	0.2869	41 (21.69)	373 (20.66)	0.8124	50 (24.87)	364 (20.30)	0.1543
Unknown	0 (0.00)	5 (0.27)	0.5307	0 (0.00)	5 (0.28)	0.4688	1 (0.50)	4 (0.22)	0.4130*
Region, *n* (%)									
Midwest	24 (16.55)	338 (18.28)	0.6832	29 (15.34)	333 (18.45)	0.3399	35 (17.41)	327 (18.24)	0.8484
Northeast	28 (19.31)	423 (22.88)	0.3759	35 (18.52)	416 (23.05)	0.1853	26 (12.94)	425 (23.70)	0.0007
South	61 (42.07)	650 (35.15)	0.1132	82 (43.39)	629 (34.85)	0.0243	87 (43.28)	624 (34.80)	0.0213
West	32 (22.07)	438 (23.69)	0.7332	43 (22.75)	427 (23.66)	0.8502	53 (26.37)	417 (23.26)	0.3693
Differential diagnosis of RTT at baseline, *n* (%)									
ASD	19 (13.10)	256 (13.85)	0.9010	14 (7.41)	261 (14.46)	0.0103	10 (4.98)	265 (14.78)	<0.0001
Angelman syndrome	0 (0.00)	3 (0.16)	0.9999*	0 (0.00)	3 (0.17)	0.9999*	0 (0.00)	3 (0.17)	0.9999*
Cerebral palsy	24 (16.55)	136 (7.36)	<0.0001	34 (17.99)	126 (6.98)	<0.0001	36 (17.91)	124 (6.92)	<0.0001
Developmental delay (unspecified)	41 (28.28)	127 (6.87)	<0.0001	39 (20.63)	129 (7.15)	<0.0001	38 (18.91)	130 (7.25)	<0.0001
Other childhood disintegrative disorder	0 (0.00)	1 (0.05)	0.9999*	0 (0.00)	1 (0.06)	0.9999*	0 (0.00)	1 (0.06)	0.9999*

ASD, autism spectrum disorder; LRTI, lower respiratory tract infection; RTT, Rett syndrome; SD, standard deviation.

* *p*-values were calculated using chi-square test or Fisher's exact test (counts <5).

**Table 2:** Baseline characteristics: incident RTT individuals with/without post-Index aspiration, LRTI, or respiratory failure.

Baseline clinical characteristics	Aspiration outcome during post-index			LRTI outcome during post-index			Respiratory failure outcome during post-index		
	Yes	No	*p*-value	Yes	No	*p*-value	Yes	No	*p*-value
	*n* = 145	*n* = 1,849		*n* = 189	*n* = 1,805		*n* = 201	*n* = 1,793	
Baseline clinical outcomes of interest, *n* (%)									
Aspiration	23 (15.86)	18 (0.97)	<0.0001	15 (7.94)	26 (1.44)	<0.0001	17 (8.46)	24 (1.34)	<0.0001
LRTI	17 (11.72)	48 (2.60)	<0.0001	43 (22.75)	22 (1.22)	<0.0001	35 (17.41)	30 (1.67)	<0.0001
Respiratory failure	15 (10.34)	56 (3.03)	<0.0001	34 (17.99)	37 (2.05)	<0.0001	61 (30.35)	10 (0.56)	<0.0001
Baseline comorbidities and grouped disorders, *n* (%)									
Cough	18 (12.41)	51 (2.76)	<0.0001	26 (13.76)	43 (2.38)	<0.0001	19 (9.45)	50 (2.79)	0.0400
Dysphagia	35 (24.14)	111 (6.00)	<0.0001	34 (17.99)	112 (6.20)	<0.0001	52 (25.87)	94 (5.24)	<0.0001
Gastrointestinal	46 (31.72)	242 (13.09)	<0.0001	62 (32.08)	226 (12.52)	<0.0001	81 (40.30)	207 (11.54)	<0.0001
Growth abnormalities/Nutritional deficiencies	21 (14.48)	106 (5.73)	<0.0001	24 (12.70)	103 (5.71)	<0.0001	34 (16.92)	93 (5.19)	<0.0001
Infections/Viruses	36 (24.83)	141 (7.63)	<0.0001	66 (34.92)	111 (6.15)	<0.0001	65 (32.34)	112 (6.25)	<0.0001
Musculoskeletal^a^	14 (9.66)	64 (3.46)	<0.0001	17 (8.99)	61 (3.38)	<0.0001	24 (11.94)	54 (3.01)	<0.0001
Scoliosis only	12 (8.28)	50 (2.70)	0.0005	16 (8.47)	46 (2.55)	<0.0001	22 (10.95)	40 (2.23)	<0.0001
Neurodevelopmental	48 (33.10)	388 (20.98)	0.0010	53 (28.04)	383 (21.22)	0.0388	61 (30.35)	375 (20.91)	<0.0001
Neurological^a^	53 (36.55)	319 (17.25)	<0.0001	71 (37.57)	301 (16.68)	<0.0001	83 (41.29)	289 (16.12)	<0.0001
Epilepsy only	43 (29.66)	251 (13.57)	<0.0001	55 (29.10)	239 (13.24)	<0.0001	67 (33.33)	227 (12.66)	<0.0001
Respiratory^a^	26 (17.93)	134 (7.25)	<0.0001	42 (22.22)	118 (6.54)	<0.0001	34 (16.92)	126 (7.03)	<0.0001
Vomiting	9 (6.21)	42 (2.27)	0.0089	14 (7.41)	37 (2.05)	<0.0001	10 (4.98)	41 (2.29)	0.0400

*P*-values were calculated using chi-square test or Fisher's exact test (counts <5); LRTI, Lower respiratory tract infection; RTT, Rett syndrome; SD, standard deviation.

^a^ Musculoskeletal includes scoliosis, kyphosis and other spinal deformities; Neurological includes seizures, epilepsy and convulsions; Respiratory includes asthma, atelectasis, COPD, breathing irregularities/abnormal breathing.

### 3.2 Rates and predictors of aspiration

**Figure 3:**
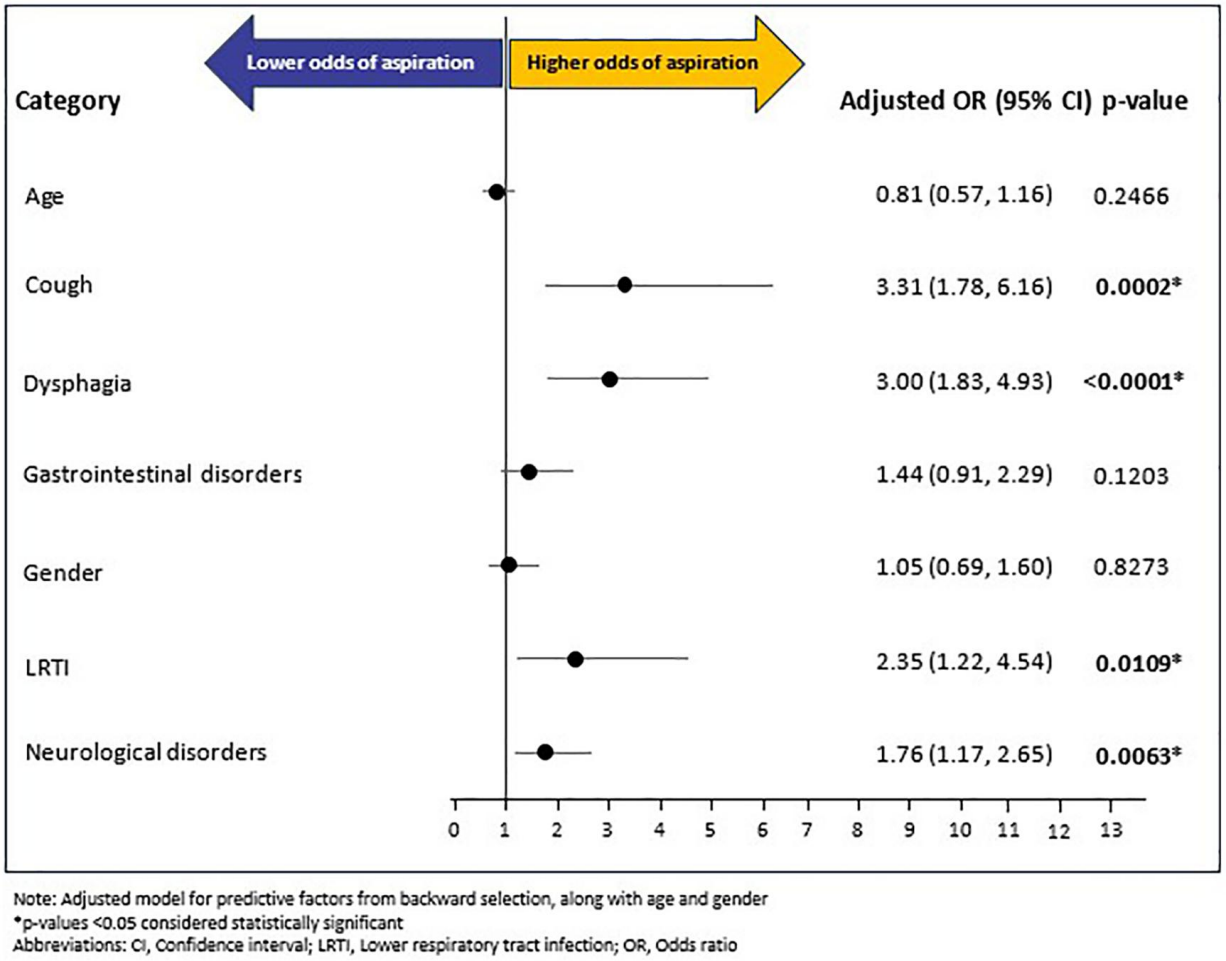
Multivariable logistic regression for aspiration.

A total of 145 (7.27%) RTT individuals experienced aspiration during the follow-up period. The mean (SD) and median (IQR) time from RTT index date to occurrence of aspiration outcome was 125 (118.44) days and 91 (221) days, respectively. Of the fourteen baseline variables (age, gender, and 12 baseline comorbidities excluding baseline aspiration) included in the backward selection regression (Supplementary Figure S1) model, five covariates emerged as potential predictors of post-index aspiration: LRTI (OR: 2.34, 95% CI: 1.22–4.51, *p* = 0.0110), cough (OR: 3.39, 95% CI: 1.82–6.29, *p* = 0.0001), dysphagia (OR: 3.04, 95% CI: 1.86–4.99, *p* < 0.0001), neurological disorders (OR: 1.77, 95% CI: 1.18–2.66, *p* = 0.0058), and gastrointestinal disorders (OR: 1.43, 95% CI: 0.90–2.26, *p* = 0.1283). Of these five, gastrointestinal disorders showed a non-significant association. The confirmatory multivariable logistic regression model, adjusted for age, gender, and the five baseline covariates (i.e., LRTI, cough, dysphagia, gastrointestinal disorders, and neurological disorders) showed that cough (OR: 3.31, 95% CI: 1.78–6.16; *p* = 0.0002), dysphagia (OR: 3.00, 95% CI: 1.83–4.93, *p* < 0.0001), LRTI (OR: 2.35, 95% CI: 1.22–4.54, *p* = 0.0109), and neurological disorders (OR: 1.76, 95% CI: 1.17–2.65, *p* = 0.0063) increased the odds of post-index aspiration multifold. In contrast, age, gender, and gastrointestinal disorders were not significantly associated with aspiration (Figure 3).

### 3.3 Rates and predictors of lower respiratory tract infection

**Figure 4:**
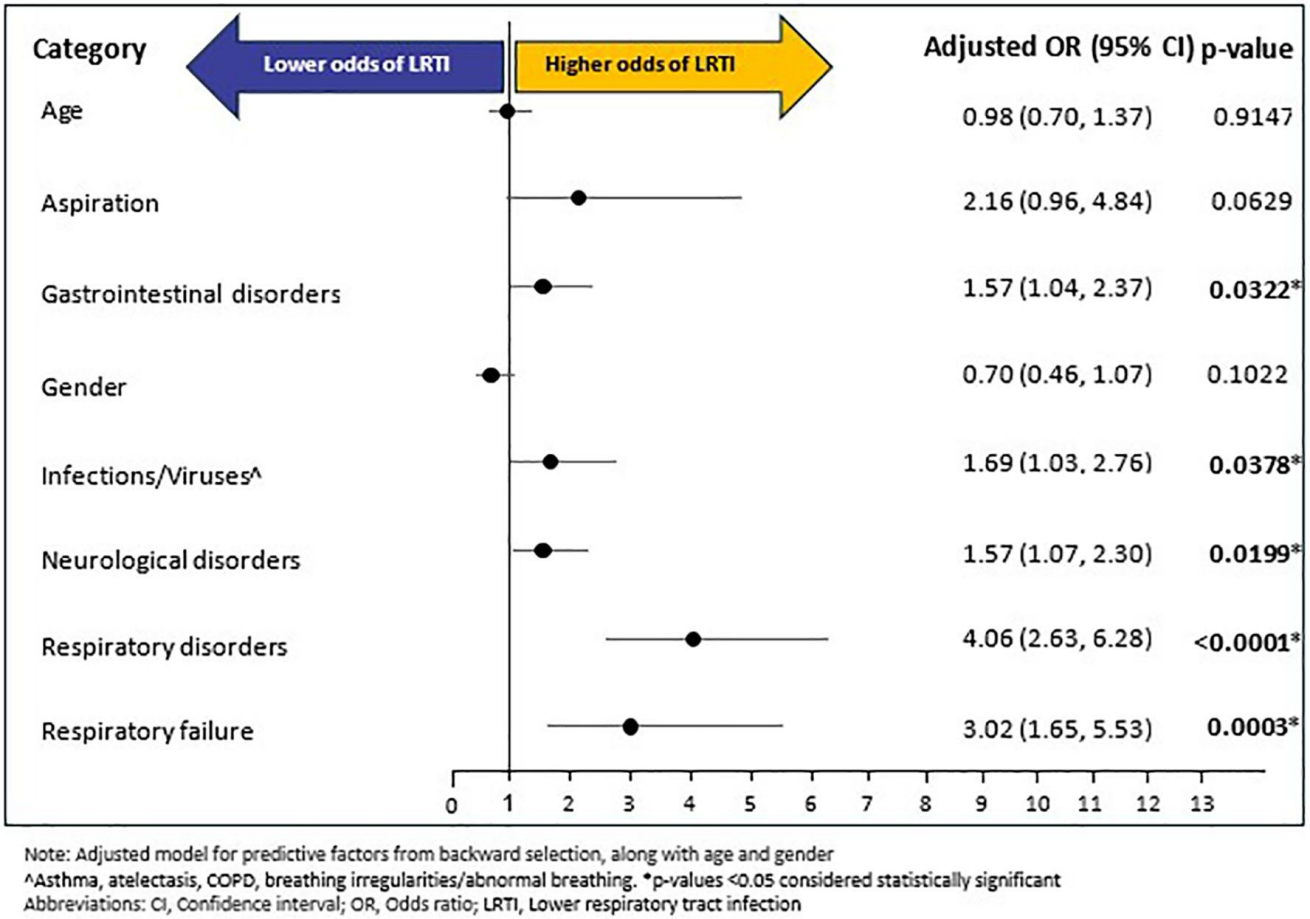
Multivariable logistic regression for LRTI.

A total of 189 (9.48%) individuals experienced LRTI during follow-up period; with a mean (SD) and median (SD) time of 124.46 (119.43) days and 91 (227) days, respectively, from RTT diagnosis to LRTI outcome. Of the fourteen baseline variables (age, gender, and 12 baseline comorbidities excluding baseline LRTI) that were included in the backward selection regression (Supplementary Figure S2), six emerged as potential predictors of LRTI during follow-up: respiratory disorders (OR: 4.06, 95% CI: 2.63–6.27, *p* < 0.0001), RF (OR: 3.02, 95% CI: 1.65–5.53, *p* = 0.0003), infections/viruses (OR: 1.69, 95% CI: 1.04–2.80, *p* = 0.0331), gastrointestinal disorders (OR: 1.57, 95% CI: 1.04–2.37, *p* = 0.0324), neurological disorders (OR: 1.57, 95% CI: 1.07–2.29, *p* = 0.0198), and aspiration (OR: 2.15, 95% CI: 0.96–4.83, *p* = 0.0635). In the confirmatory multivariable logistic regression, adjusted for age and gender, and predictive factors of backward selection, five of the six predictors from the backward selection model remained as significant predictors of LRTI: respiratory disorders (OR: 4.06, 95% CI: 2.63–6.28, *p* < 0.0001), RF (OR: 3.02, 95% CI: 1.65–5.53, *p* = 0.0003), neurological disorders (OR: 1.57, 95% CI: 1.07–2.30, *p* = 0.0199), infections/viruses (OR: 1.69, 95% CI: 1.03–2.76, *p* = 0.0378) and gastrointestinal disorders (OR: 1.57, 95% CI: 1.04–2.37, *p* = 0.0322). On the other hand, age, gender, and baseline aspiration were not significantly associated with LRTI (Figure 4).

### 3.4 Rates and predictors of respiratory failure

**Figure 5:**
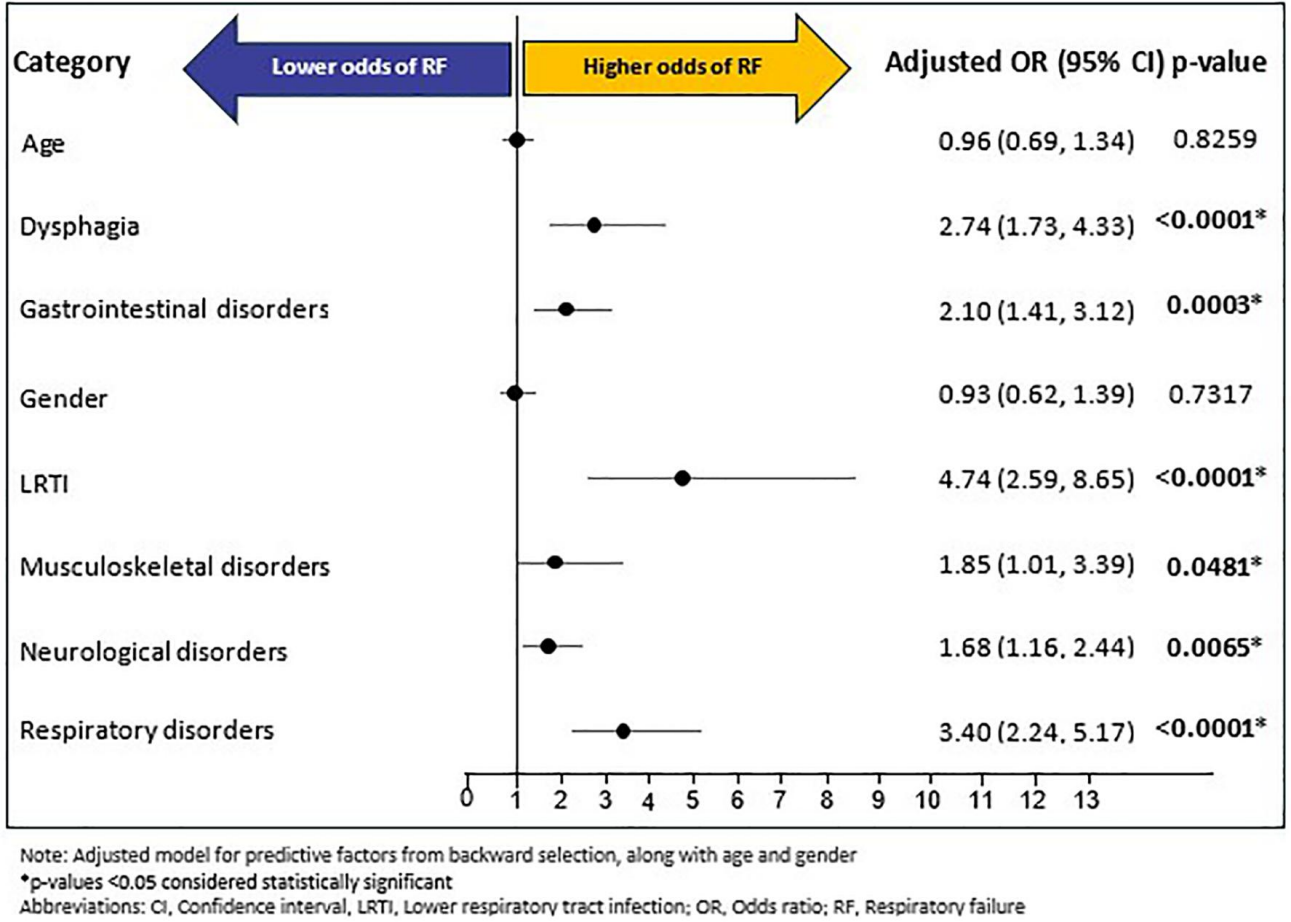
Multivariable logistic regression for respiratory failure.

A total of 201 (10.08%) of individuals experienced RF during follow-up period; with a mean (SD) and median (IQR) time of 105.95 (120.17) days and 42 (215) days, respectively, from index RTT diagnosis to RF outcome. Of the fourteen baseline predictors (age, gender, and 12 baseline comorbidities excluding baseline RF) included in the backward selection regression, six were predictive of RF during follow-up (Supplementary Figure S3): LRTI (OR: 4.70, 95% CI: 2.58–8.58, *p* < 0.0001), respiratory disorders (OR: 3.38, 95% CI: 2.23–5.13, *p* < 0.0001), dysphagia (OR: 2.73, 95% CI: 1.73–4.31, *p* < 0.0001), gastrointestinal disorders (OR: 2.09, 95% CI: 1.40–3.10, *p* = 0.0003), musculoskeletal disorders (OR: 1.86, 95% CI: 1.01–3.40, *p* = 0.0459), and neurological disorders (OR: 1.69, 95% CI: 1.16–2.45, *p* = 0.0061). In the confirmatory multivariable logistic regression model, adjusted for age, gender, and predictive factors of backward selection, all six predictors identified in the backward selection remained as significant predictors of RF: LRTI (OR: 4.74, 95% CI: 2.59–8.65, *p* < 0.0001), respiratory disorders (OR: 3.40, 95% CI: 2.24–5.17, *p* < 0.0001), dysphagia (OR: 2.74, 95% CI: 1.73–4.33), *p* < 0.0001), gastrointestinal disorders (OR: 2.10, 95% CI: 1.41–3.12, *p* = 0.0003), musculoskeletal disorders (OR: 1.85, 95% CI: 1.01–3.39, *p* = 0.0481), and neurological disorders (OR: 1.68, 95% CI: 1.16–2.44, *p* = 0.0065). Age and gender were not significantly associated with RF (Figure 5).

In a sensitivity analysis of the models in which the baseline rates of the specific outcome were also included as a covariate, the models showed the baseline occurrence of the outcome is the single biggest predictor for each of the outcomes of interest.

## 4 Discussion

To date, no studies in RTT have examined external factors that predict the occurrence of respiratory outcomes such as aspiration, LRTI, and RF, respectively. This claims database analysis is the first of its kind to evaluate real-world occurrence of aspiration, LRTI, RF and their associated predictors. In this analysis, 1 in 10 individuals with RTT experienced one or more of the three respiratory outcomes: aspiration, LRTI, or RF during the 12-month follow-up period. On average, aspiration or LRTI occurred approximately 4-months from index-date while RF occurred approximately 3.5 months from index date. Our analysis also found that baseline history of specific clinical comorbidities were independent predictors of post-index manifestation of respiratory complications while age and gender were not associated with an increased odds of any of the outcomes of interest (aspiration, LRTI, and RF). Specifically, the likelihood of aspiration after RTT diagnosis was threefold higher among individuals with a history of either cough or dysphagia, while it was twofold higher among those with a history of LRTI or neurological disorders (i.e., epilepsy, convulsions). Biological mechanisms linking these predictors with an increased likelihood of aspiration may involve both physiological and neurological pathways (4, 20). For instance, dysphagia and cough may directly contribute to the misdirection of food and liquids into the airway, subsequently increasing the odds of aspiration (20). While it is not surprising to observe that the history of LRTI (i.e., pneumonia) is an independent predictor of aspiration, history of neurological disorders (i.e., composite of epilepsy, convulsions, or seizures) as a significant predictor of aspiration is an interesting finding in current research.

Similarly, the history of respiratory disorders, RF, neurological disorders, infections/viruses, and gastrointestinal disorders were significant predictors of LRTI occurrence after RTT diagnosis. Respiratory disorders had the highest explanatory power in predicting LRTI, followed by RF; with the likelihood of LRTI being increased more than four times and three times, respectively. The greater likelihood of LRTI among individuals with RTT who have history of respiratory disorders such as asthma, or breathing abnormalities appears to align with published literature that shows higher LRTI rates among RTT individuals with asthma or breathing abnormalities such as breath holding or hyperventilation (21). There was a higher likelihood of LRTI among those with a history of neurological disorders and gastrointestinal disorders, which requires further investigation. Likewise, the history of LRTI and respiratory disorders increased the likelihood of RF nearly fivefold, and threefold, respectively. Additionally, dysphagia contributed to a nearly threefold likelihood of RF, while neurological, musculoskeletal, and gastrointestinal disorders increased the likelihood of RF approximately two-fold.

Our findings align with existing research emphasizing the susceptibility of individuals with RTT to respiratory complications due to aspiration, silent aspiration, and feeding difficulties and dysphagia (8, 22). Silent aspiration, identified in a substantial proportion of individuals with RTT, often leads to recurrent LRTI and increases the risk of RF. Additionally, published literature highlights the complex interplay between feeding dysfunctions and respiratory health in RTT (23, 24). Specifically, the endoscopic evaluation by Sideris et al., using fiberoptic endoscopic swallowing evaluations found that aspiration incidents occurred primarily during the pre-pharyngeal phase, with pureed foods being strongly associated with LRTI such as pneumonia, compared to liquids. Furthermore, mobility plays a critical protective role in respiratory complications, with independent walking significantly reducing the risk of respiratory complications compared to assisted walking or immobility (13, 21). Finally, these findings highlight the intricate relationship between respiratory, neurological, and gastrointestinal disorders with the occurrence of LRTI and RF, while dysphagia, and musculoskeletal disorders (i.e., scoliosis, kyphosis, and other spinal deformities) remain as additional independent predictors of RF.

While our primary analysis identified external predictors of the outcomes of interest, the sensitivity analysis suggests that baseline occurrence would be the single biggest predictor of the post-index outcome in any scenario. Therefore, it is important to note that individuals who already suffer from these manifestations at baseline may require specialized attention, and specific treatment and management strategies that can alleviate these occurrences will need to be designed before the initiation of treatment and management of RTT.

In our study, 9.5% of RTT patients experienced LRTI, a lower rate than the 21%–22% reported in large cohorts (15). MacKay et al. similarly found 21.4% hospitalized for LRTI, with risk linked to clinical factors: enteral feeding nearly doubled risk and non-ambulatory status increased it sixfold, highlighting the protective role of mobility and optimized feeding. Aspiration (7.3%) was also lower than prior reports (14%–28%), whereas respiratory failure (10.1%) was slightly higher than the 6%–7% observed in surveillance study but below high-risk subgroups (8). These differences likely reflect variations in study design, data source, and population characteristics.

Although our study examined the annual rates of aspiration, LRTI, and RF following RTT diagnosis as well as associated baseline predictors, longitudinal studies of individuals with RTT could provide valuable insights into the progression of respiratory complications and help identify critical periods for intervention. Such studies would also help validate the findings of this research and provide supporting evidence for the generation of evidence-based RTT treatment and management guidelines.

Several etiological hypotheses may help to explain the associations we identified. Ramirez et al. proposed that MECP2 deficiency disrupts central autonomic and respiratory neural networks, contributing to unstable breathing patterns, recurrent apneas, and increased vulnerability to infections (25). Additionally, it is possible the co-occurrence of gastrointestinal and neurological comorbidities observed in our cohort may exacerbate respiratory risks through mechanisms such as aspiration, impaired airway clearance, and autonomic dysregulation. Dysphagia and impaired swallowing coordination can further exacerbate aspiration risk, contributing to a cycle of infections and compromised respiratory function (26). These biological and clinical pathways warrant further investigation in longitudinal and clinical studies.

Recent clinical consensus has translated this evidence into practical guidelines for RTT management. The Italian Delphi panel (27) recommends routine respiratory screening for disordered breathing while awake and during sleep, careful monitoring of swallowing function, and early pulmonology referral for recurrent infections, hypoxemia, or hypoventilation. Recommended surveillance tools include sleep studies, pulse oximetry, blood gases, and chest imaging. While preventive interventions include vaccination, airway clearance, reflux and scoliosis management, and nutritional support, with CPAP/BiPAP or tracheostomy reserved for severe dysfunction. Caregiver education and multidisciplinary coordination are emphasized as essential for long-term outcomes.

The burden of respiratory complications in RTT extends beyond clinical impact to quality of life and healthcare resource use. Frequent respiratory infections, prolonged hospital stays, and the need for interventions such as ventilatory support or enteral feeding add considerable stress for families and contribute to rising healthcare costs (28, 29). An observational study indicated that respiratory illness is among the most common reasons for hospitalization in RTT, often necessitating resource-intensive interventions (15). As respiratory function declines over time, individuals face greater likelihood of depending on non-invasive ventilation, experiencing recurrent aspiration, and having increased risk of premature mortality, all of which highlight the need for proactive and ongoing management (15, 28).

Overall, the findings of our study have important clinical implications that extend beyond respiratory complications. First, the identification of baseline predictors provides important insights into the multifaceted relationship involving the different organ systems (i.e., neurological, gastrointestinal, musculoskeletal) and the core symptoms of RTT (i.e., feeding difficulties, dysphagia, scoliosis) that are associated with the occurrence of respiratory manifestations of aspiration, LRTI, and RF. Second, results of this current study sheds new light on the potential role of neurological manifestations on respiratory outcomes (and also provides additional insights into the role of musculoskeletal conditions on the occurrence of RF. Third, these insights suggest the need to help develop targeted intervention and management strategies among individuals with various baseline risk factors to mitigate the likelihood of occurrence of these debilitating respiratory manifestations. Furthermore, these findings suggest that a baseline history of respiratory manifestations was the strongest predictor of subsequent respiratory events. These findings may help design apriori treatment protocols tailored for such individuals to reduce occurrence in the post-RTT diagnosis period. Finally, our findings are corroborated by published research that suggests the need for vigilant monitoring, multidisciplinary approach to managing RTT, individualized treatment protocols, and proactive clinical practice management of individuals with RTT (4, 21, 30).

## 5 Limitations

As with any real-world data analysis, our study has several limitations. The use of retrospective claims data may introduce potential biases, such as misclassification of diagnosis or incomplete data capture due to miscoding or under coding issues. Although RTT is a rare disease, the generalizability of the results may be limited by the study's reliance on a specific dataset, which may not fully represent the broader RTT population. It is also possible that unobservable socioeconomic factors not available in claims databases may confound the results further. These limitations suggest that while the findings are robust, they should be interpreted with caution.

While this study examined newly diagnosed individuals, a key limitation of our study design is that the 6-month washout period cannot reliably capture truly incident RTT cases. The mean age at diagnosis in our dataset (21 years) which is substantially older than the expected age of clinical diagnosis (2–3 years), suggesting that many cases reflect delayed coding or incident claims coding rather than incident diagnoses. We understand it is plausible for the rates of new onset or incident cases may not be reliably identified with shorter washout periods. However, rare conditions such as RTT are identified through a process of differential diagnosis (i.e., other related conditions are usually ruled out); thus, reducing the limitations of inaccurate diagnosis. While our findings may represent patterns of morbidity following the first claims-based recognition of RTT rather than the biological onset of disease, we believe that the RTT patients in our analysis may be reflective of true positive cases of RTT.

Finally, because many RTT symptoms precede formal diagnosis, baseline comorbidities such as aspiration or LRTI may represent early manifestations of RTT rather than independent predictors, raising the possibility of reverse causation and constraining causal inference in retrospective claims data (14). As a result, claims data can only show associations, not causation. Future studies using time-dependent covariate analyses with longer time and wash-out periods, or clinical trials may better capture the chronic and progressive nature of RTT and clarify temporal relationships between predictors and outcomes.

## 6 Conclusion

Published research in RTT has shown that the history of respiratory complications may be a predictive risk factor for the occurrence of aspiration, LRTI, or RF during follow-up and post-RTT diagnosis. However, this is the first study to quantify the magnitude of predictive risk associated with baseline comorbidities and the occurrence of aspiration, LRTI, and RF. Additionally, this analysis also showed that the history of gastrointestinal disorders is a significant risk factor for the occurrence of LRTI and RF after diagnosis of RTT. Furthermore, this study is the first in the literature to demonstrate that neurological disorders (i.e., epilepsy, seizures) may be a risk factor for all three respiratory outcomes of interest: aspiration, LRTI, and RF. These surprising, yet important findings require further investigation to understand the biological relationship between neurological disorders and the occurrence of these respiratory outcomes. Lastly, the history of musculoskeletal conditions was found to be a risk factor for the occurrence of RF after RTT diagnosis.

## Data Availability

The datasets presented in this article are not readily available because the data sources used in this analysis are not publicly available, and the databases are syndicated and available upon use with a data license fee. Requests to access the datasets should be directed to Krithika Rajagopalan, kr.rajagopalan@anlitiks.com.
