# Supplementary material for: Predictors of aspiration, lower respiratory tract infection, and respiratory failure among individuals with Rett Syndrome: analysis of real-world claims data in the United States
Source: Front Pediatr. 2025 Oct 20;13:1681103. doi: 10.3389/fped.2025.1681103 (PMC12580911; doi:10.3389/fped.2025.1681103)
Supplement: Supplementary file 1 [file Datasheet1.docx]

**Supplementary** **Material**

**Table S1: ICD-10-CM Codes for Baseline Comorbidities and Clinical Outcomes of Interest**

| **Condition Name** | **ICD-10 CODES** |
| --- | --- |
| Aspiration | J69.0, J69.8, Y84.4, 92610, 74230 |
| Cough | R05.X |
| Dysphagia | R13.1X |
| Lower respiratory tract infection | J09.X1, J10.0X, J12.X-J18.X, J20.X-J22.X, J40.X |
| Respiratory failure | J80, J95.82X, J96.XX, R09.02, R09.2, V46.1X, V46.2, Z99.11, Z99.81,  E0601 |
| Vomiting | R11.1X, R11.2 |

ICD-10-CM: International Classification of Diseases, Tenth Revision, Clinical Modification

**Table S2: ICD-10-CM Codes for Differential Diagnosis**

| **Condition Name** | **ICD-10 CODES** |
| --- | --- |
| Autism spectrum disorder | F84.0 |
| Other childhood disintegrative disorder | F84.3 |
| Cerebral palsy | G80 |
| Angelman syndrome | Q93.51 |
| Non-specific developmental delay | R62.50 |

ICD-10-CM: International Classification of Diseases, Tenth Revision, Clinical Modification

**Table S3: ICD-10-CM Codes for Baseline Comorbidities Grouped into Disorders**

| **Grouped Disorders** | **Description** | **ICD-10 CODES** |
| --- | --- | --- |
| Gastrointestinal disorders | Constipation, diarrhea, gallbladder dysfunction, gastroesophageal reflux disorder, gastrostomy, gastroparesis | 009.2, 009.3, 564.5, 787.91, K21.XX, K31.84, K58.9, K59.1, K59.0X, K80.XX-K82.XX, K87, K94.2X, P78.3, R19.7, Z93.1 |
| Growth abnormalities/  Nutritional disorders | Underweight, short stature, nutritional deficiency and failure to thrive | E40-E46, E63.X, R62.51, R62.7, R62.52, R62.59, R63.6 |
| Infectious/Viruses | COVID-19, respiratory syncytial virus, influenza, fever, upper respiratory infection | B34.X, B97.X, J00.X-J06.X, J09.X2, J09.X3, J09.X9, J10.X, J11.X,  J20.5, N39.0, R30.0, R50.9, U00, U09, U49, Z87.440 |
| Musculoskeletal disorders | Kyphosis, and other spinal deformities | 754.2, M40.05, M40.14, M40.204, M40.209, M40.57, M41.XX, M43.9, Q67.5, Q76.414, R29.3 |
| Scoliosis only | Scoliosis | M41.XX |
| Neurodevelopmental disorders | Behavioral disorders and disturbance symptoms^#^ movement disorders, development progress delayed, microcephaly, loss of acquired communication skills, loss of acquired motor skills, weakness/paralysis, prominent hand apraxia/dyspraxia, wasting, dystonia, bradykinesia, sleep dysfunction | 343.2, F51.01, F51.04, F80.XX, F82, F90.X, F91.X, F94.X, F95.X, F98.XX, F98.4, G24.1–G24.5, G24.8, G24.9, G25.5, G25.9, G26, G47.0X-G47.2X, G47.4-G47.6, G47.61, G47.8, G47.9, G82.50, M62.5X, M62.81, P94.2, Q02, R25.8, R27.8, R45.1, R45.4-R45.6, R45.83, R48.2, R62.0, |
| Neurological disorders | Epilepsy , convulsions | R56.XX, G40.XX |
| Epilepsy only | Epilepsy | G40.XX |
| Respiratory disorders | Asthma, atelectasis, COPD, breathing irregularities/abnormal breathing | G47.3, J440, J441, J449, J45.XX, J98.11, P28.4X, R06.0X, R06.1, R06.2, R06.3, R06.4, R06.89, R08.81, R08.82, R08.89, R08.9 |

# Irritability, aggressive behavior, crying tantrums, self-mutilation, scratching, biting

Abbreviations: ICD-10-CM, International Classification of Diseases, 10th Revision, Clinical Modification

**Table S4: Baseline Demographics & Characteristics of Study Outcomes for Incident RTT Individuals (Unadjusted Odds Ratios)**

| **Baseline Demographics and Clinical Characteristics** | **Aspiration outcome**  **during post-index** | | | | **LRTI outcome**  **during post-index** | | | | **Respiratory failure outcome**  **during post-index** | | | |
| --- | --- | --- | --- | --- | --- | --- | --- | --- | --- | --- | --- | --- |
|  | **Yes** | **No** | **OR***  **(95% CI)** | **p-value** | **Yes** | **No** | **OR***  **(95% CI)** | **p-value** | **Yes** | **No** | **OR***  **(95% CI)** | **p-value** |
|  | **n=145** | **n=1849** |  |  | **n=189** | **n=1805** |  |  | **n=201** | **n=1793** |  |  |
| **Age (<18 yrs)** | 82 (56.55%) | 937 (50.68%) | 1.27  (0.90, 1.78) | 0.2017 | 98 (51.85%) | 921 (51.02%) | 1.03  (0.77, 1.40) | 0.8887 | 105 (52.24%) | 914 (50.98%) | 1.05  (0.79, 1.41) | 0.7908 |
| **Gender (Male)** | 36  (24.83%) | 378 (20.44%) | 1.28  (0.86, 1.90) | 0.2514 | 41 (21.69%) | 373 (20.66%) | 1.06  (0.74, 1.53) | 0.8124 | 50 (24.88%) | 364 (20.30%) | 1.3  (0.93, 1.83) | 0.1543 |
| **Aspiration** | 23 (15.86%) | 18  (0.97%) | 19.18  (10.08, 36.49) | <0.0001 | 15  (7.94%) | 26  (1.44%) | 5.9  (3.07, 11.35) | <0.0001 | 17  (8.46%) | 24  (1.34%) | 6.81  (3.59, 12.91) | <0.0001 |
| **Cough** | 18 (12.41%) | 51  (2.76%) | 5 (2.84, 8.81) | <0.0001 | 26 (13.76%) | 43  (2.38%) | 6.54  (3.91, 10.91) | <0.0001 | 19 (9.45%) | 50 (2.79%) | 3.64  (2.10, 6.31) | <0.0001 |
| **Dysphagia** | 35 (24.14%) | 111 (6.00%) | 4.98  (3.25, 7.63) | <0.0001 | 34 (17.99%) | 112 (6.20%) | 3.32  (2.18, 5.03) | <0.0001 | 52 (25.87%) | 94  (5.24%) | 6.31  (4.32, 9.20) | <0.0001 |
| **Gastrointestinal disorders** | 46 (31.72%) | 242 (13.09%) | 3.09  (2.12, 4.49) | <0.0001 | 62 (32.80%) | 226 (12.52%) | 3.41  (2.44, 4.76) | <0.0001 | 81 (40.30%) | 207 (11.54%) | 5.17  (3.77, 7.10) | <0.0001 |
| **Growth abnormalities/**  **nutritional disorders** | 21 (14.48%) | 106 (5.73%) | 2.78  (1.69, 4.60) | <0.0001 | 24 (12.70%) | 103 (5.71%) | 2.4  (1.50, 3.85) | <0.0001 | 34 (16.92%) | 93  (5.19%) | 3.72  (2.44, 5.69) | <0.0001 |
| **Infectious/**  **Viruses** | 26 (17.93%) | 134 (7.25%) | 2.8  (1.77, 4.43) | <0.0001 | 42 (22.22%) | 118 (6.54%) | 4.08  (2.76, 6.04) | <0.0001 | 34 (16.92%) | 126 (7.03%) | 2.69  (1.79, 4.06) | <0.0001 |
| **LRTI** | 17 (11.72%) | 48  (2.60%) | 4.98  (2.79, 8.91) | <0.0001 | 43 (22.75%) | 22  (1.22%) | 23.87  (13.90, 40.99) | <0.0001 | 35 (17.41%) | 30  (1.67%) | 12.39  (7.42, 20.7) | <0.0001 |
| **Musculoskeletal disorders** | 14  (9.66%) | 64  (3.46%) | 2.98  (1.63, 5.46) | <0.0001 | 17 (8.99%) | 61 (3.38%) | 2.83  (1.61, 4.95) | <0.0001 | 24 (11.94%) | 54  (3.01%) | 4.37  (2.64, 7.24) | <0.0001 |
| **Neuro-developmental disorders** | 48 (33.10%) | 388 (20.98%) | 1.86  (1.30, 2.68) | <0.0001 | 53 (28.04%) | 383 (21.22%) | 1.45  (1.03, 2.03) | 0.0388 | 61 (30.35%) | 375 (20.91%) | 1.65  (1.19, 2.27) | <0.0001 |
| **Neurological disorders** | 53 (36.55%) | 319 (17.25%) | 2.76  (1.93, 3.96) | <0.0001 | 71 (37.57%) | 301 (16.68%) | 3.01  (2.18, 4.14) | <0.0001 | 83 (41.29%) | 289 (16.12%) | 3.66  (2.69, 4.98) | <0.0001 |
| **Respiratory disorders** | 36 (24.83%) | 141 (7.63%) | 4  (2.64, 6.05) | <0.0001 | 66 (34.92%) | 111 (6.15%) | 8.19  (5.74, 11.68) | <0.0001 | 65 (32.34%) | 112 (6.25%) | 7.17  (5.04, 10.20) | <0.0001 |
| **Respiratory failure** | 15 (10.34%) | 56  (3.03%) | 3.69  (2.03, 6.71) | <0.0001 | 34 (17.99%) | 37  (2.05%) | 10.48  (6.40, 17.17) | <0.0001 | 61 (30.35%) | 10  (0.56%) | 77.69  (38.95, 154.90) | <0.0001 |
| **Vomiting** | 9  (6.21%) | 42  (2.27%) | 2.85  (1.36, 5.97) | 0.0089 | 14  (7.41%) | 37  (2.05%) | 3.82  (2.03, 7.21) | <0.0001 | 10  (4.98%) | 41  (2.29%) | 2.24  (1.10, 4.54) | 0.0400 |

*Unadjusted odds ratio; Abbreviations: LRTI, Lower respiratory tract infection; OR, Odds ratio; CI, Confidence interval

**Figure S1. Backward Selection Logistic Regression for Aspiration**

**Category**

Cough

Dysphagia

Gastrointestinal disorders

LRTI

Neurological disorders

**Adjusted OR (95% CI)**

3.39 (1.82, 6.29)

3.04 (1.86, 4.99)

1.43 (0.90, 2.26)

2.34 (1.22, 4.51)

1.77 (1.18, 2.66)

**p-value**

**0.0001***

<**0.0001***

0.1283

**0.0110***

**0.0058***

1

2

3

4

5

6

7

**Lower odds of aspiration**

**Higher odds of aspiration**

8

9

10

11

12

13

Note: Variables retained after removing baseline aspiration

*p-values <0.05 considered statistically significant

Abbreviations: CI, Confidence interval; LRTI, Lower respiratory tract infection; OR, Odds ratio

0

**Figure S2. Backward Selection Logistic Regression for LRTI**

**Category**

Aspiration

Gastrointestinal disorders

Gender

Infections/Viruses^

Neurological disorders

Respiratory disorders

Respiratory failure

**Adjusted OR (95% CI)**

2.15 (0.96, 4.83)

1.57 (1.04, 2.37)

0.70 (0.46, 1.07)

1.69 (1.04, 2.80)

1.57 (1.07, 2.29)

4.06 (2.63, 6.27)

3.02 (1.65, 5.53)

**p-value**

0.0635

**0.0324***

0.1021

**0.0331***

**0.0198***

<**0.0001***

**0.0003***

1

2

3

4

5

6

7

**Lower odds of LRTI**

**Higher odds of LRTI**

8

9

10

11

12

13

0

Note: Variables retained after removing baseline LRTI

^Asthma, atelectasis, COPD, breathing irregularities/abnormal breathing. *p-values <0.05 considered statistically significant

Abbreviations: CI, Confidence interval; LRTI, Lower respiratory tract infection; OR, Odds ratio

**Figure S3. Backward Selection Logistic Regression for Respiratory Failure**

**Category**

Dysphagia

Gastrointestinal disorders

LRTI

Musculoskeletal disorders

Neurological disorders

Respiratory disorders

**Adjusted OR (95% CI)**

2.73 (1.73, 4.31)

2.09 (1.40, 3.10)

4.70 (2.58, 8.58)

1.86 (1.01, 3.40)

1.69 (1.16, 2.45)

3.38 (2.23, 5.13)

**p-value**

<**0.0001***

**0.0003***

<**0.0001***

**0.0459***

**0.0061***

<**0.0001***

1

2

3

4

5

6

7

**Lower odds of RF**

**Higher odds of RF**

8

9

10

11

12

13

14

15

0

Note: Variables retained after removing baseline RF

*p-values <0.05 considered statistically significant

Abbreviations: CI, Confidence interval; OR, Odds ratio; LRTI, Lower respiratory tract infection; RF, respiratory failure
